# Rest Rust ! Physical active for active and healthy ageing

**Published:** 2016-01-31

**Authors:** M Vollenbroek-Hutten, S Pais, S Ponce, M Dekker-van Weering, S Jansen-Kosterink, F Schena, N Tabarini, F Carotenuto, V Iadicicco, M Illario

**Affiliations:** 1 Roessingh Research and Development, Telemedicine group, Enschede, The Netherlands; 2 University of Twente, Faculty of Electrical Engineering, Mathematics and Computer Science, Telemedicine group, Enschede, The Netherlands; 3 Center for Research and Development in Health, University of Algarve, Portugal; 4 Kronikgune, Torre del BEC, Ronda de Azkue, 1, 48902 Barakaldo, Biscay, Spain; 5 Department of Neurological, Biomedical and Movement Sciences – University of Verona, Italy; 6 Department of Translational Medical Sciences, Federico II University, and R&D Unit, Federico II University Hospital

**Keywords:** physical activity, physical capacity, older adults

## Abstract

The aim of this paper is to give an insight on how physical activity can be defined, parameterized and measured in older adults and on different options to deal with citizen physical activity promotion at European level. Three relevant aspects are highlighted:

1. When talking about physical activity, two different aspects are often unfairly mixed up: “physical activity” and “physical capacity”.
  - Physical activity, is referred to as the level of physical activity someone **is actually performing** in daily life.
  - Physical capacity is referred to as **the maximum physical activity a person can perform**.
2. Both physical activity and physical capacity can be expressed in different dimensions such as time, frequency, or type of activity with the consequence that there are many tools and techniques available. In order to support people to choose an appropriate instrument in their everyday practice a list of 9 criteria that are considered important is defined.
3. Older adults score differently across the various physical dimensions, so strategies to promote physical activity should consider individual differences, in order to adapt for these variations.

## I. THE IMPORTANCE OF PHYSICAL ACTIVITY IN TO RELATION ACTIVE AND HEALTHY AGEING

Demographic ageing is a global trend. In the European Union, the number of people aged 65+ will almost double over the next 50 years, from 85 million in 2008 to 151 million in 2060. Among older adults, frailty is highly prevalent and constitutes a major health problem. Frail individuals are vulnerable and at high risk of adverse health outcomes. They have functional impairments which often result in falls, immobility and confusion. People affected by frailty are key community resource users, such as, hospitals and long-term care institutions [1].

Adopting a healthy lifestyle, is considered to be one of the key aspects to help older adults to improve their functional level and delay frailty. Although there is not a single clear definition a “healthy lifestyle”, it can be considered the steps, actions and strategies an individual puts in place to achieve optimum health. Several advantages of becoming physically active are currently acknowledged. Being physically active along with healthy eating, emotional and spiritual wellness are generally considered relevant aspect of health. Thus increasing physical activity is a potential important strategy to avoid disease and disability, maintain physical and cognitive function and engagement in social and productive activities all of which contribute for a successful aging among older adults [2].

Regular physical activity has been shown to be successful against various components of frailty in older people of both sexes, including functional impairment, cognitive and depressive performance [3], therefor ongoing participation in physical activity is important and necessary for older people [4,5]. Despite these many well-known benefits of physical activity, studies have also demonstrated that the vast majority of older adults are physically inactive and that the prevalence of inactivity increases with the advance of age [6–8]. As a consequence, there is an increasing focus on studies and initiatives that develop successful strategies to support older adults to get physically active. Both literature, and commitments brought together within the European Innovation Partnership on Active and Healthy Aging (EIP-AHA), show that these studies and initiatives can be roughly distinguished in two types of approaches:

1. Those that focus on getting insight in the level of physical activities of general older adults or in specific sub-groups with chronic diseases. These provide insight on what exactly is the physical activity in older adults or sub-groups, the variability, its deficit and consequences. Increasing this knowledge is considered to be an important first step to define what successful strategies may be used to increase physical activity level in this population.
2. Those that focus on the development and evaluation of interventions aimed at promoting physical activity either using more standard forms such as, face to face exercise classes or individual therapy, or other alternative interventions focusing on enhancing self management, home based or using new technologies used to support and increase physical activity in older adults or among these in specific subgroups.

Despite the growing attention to this aspect in elderly, it is still very difficult for professionals working in this field, to have access to a good overview to support decisions on what instruments, type of exercises or strategies to use in either research or daily practice.

The definition of physical activity itself is another difficulty, once across studies or initiatives different concepts are being used. In addition, physical activity is being expressed in a variety of parameters and assessed with different tools and instruments. Also, different standards of adequate levels of physical activity in older adults are referred.

The aim of this paper is to give clinicians, researchers and policy makers more insight in how physical activity can be defined, measured and parameterized in older adults and give some insights on how to deal with physical activity promotion among elderly citizens at a European level.

## II WHAT IS PHYSICAL ACTIVITY AND WHAT ARE THE CURRENT NORMS?

There are several definitions of physical activity. Although these definitions are comparable there are some subtle differences. The most used definition is the definition of Caspersen (1985)[9]. He defined physical activity as:

“any bodily movement produced by skeletal muscles that requires energy expenditure”.

The American College of Sports Medicine made the definition a bit more specific by adding a remark about resting energy expenditure. They (ACSM 2009) [10] and the WHO [11] define physical activity in a similar way as:

“any bodily movement produced by the contraction of skeletal muscles that result in a substantial increase in caloric requirements over resting energy expenditure”

Starting from this definition there are recommendations and norms giving for physical activity in adults by different organizations (ACSM, Dutch norm, the United States department of health and human services (HHS), WHO). For example:

- The American College of Sports Medicine recommends that the majority of adults perform moderate-intensity cardio respiratory exercise training for at least thirty minutes a day [10].
- The Dutch norm for healthy physical activity (NNGB)[12] for adults applies a physical activity for at least half an hour per day, with a minimum of five days a week on moderate-to-vigorous physical activity (MVPA) level (≥ 4 MET, Metabolic Equivalents of Task).
- The United States Department of Health and Human Services (HHS) defined the Activity Advisory Committee Report in 2008 [13]. This report indicates a physical activity norm of 150 minutes of MVPA a week.
- WHO developed the “Global Recommendations on Physical Activity for Health” [11] with the overall aim of providing national and regional level policy makers, with guidance on the dose-response relationship between the frequency, duration, intensity, type and total amount of physical activity needed for the prevention of NCDs. The recommendations address three age groups: 5–17 years old; 18–64 years old; and 65 years old and above for who recommendations are of at least 30 minutes of MVPA (≥ 3 MET) for minimally of five days a week.
- Among others, the U.S. Department of Health & Human Services [14] provide additional recommendations specific for older adults:
  - When unable to do 150 minutes of moderate-intensity aerobic activity a week due to chronic conditions, older adults should be as physically active as their abilities and conditions allow.
  - Understand whether and how their chronic conditions may affect their ability to safely do regular physical activity.
  - When with increased falling risk older adults should exercise to maintain or improve balance.
  - Effort level relative to fitness level should be determined.

Although these recommendations and norms show small differences, all express physical activity in a level (duration, intensity) that someone should deploy during a certain moments of time (day, week).

In some literature physical activity is often mixed up with physical capacity. Physical Activity is mainly referred to, as the amount of activity a subject performs on a daily life bases. In contrast physical capacity is referred to as the maximum ability that a person can perform. Physical capacity is often used in clinical practice to assess whether someone has a deficit in physical function and is often used as an outcome parameter to evaluate changes after a physical activity intervention strategy. Promoting physical activity is often seen as a strategy to improve physical capacity. Thus these two terms are strongly related. In the remainder of this paper physical capacity and physical activity are being addressed as two separate aspects as both being important to improve physical health status.

## III WHAT TOOLS AND TECHNIQUES TO USE AND HOW TO MEASURE PHYSICAL ACTIVITY?

Physical activities can be performed in different ways. It can be executed via unstructured activities incorporated in daily life (daily activity) or via structured planned activities (like sports or exercises classes) and can, as becomes clear from the recommendations, be expressed in different key dimensions like frequency, intensity, time, and type of activity. As a result of this variation there are several tools and techniques that are currently being used to assess physical activity. Generally two different methods can be discerned: subjective (self-reported) and objective measures of physical activity. Both have advantages and disadvantages which are presented below.

### Self-report measures of physical activity

Physical activity is often assessed using self-report measures [6]. These methods are easy to administer and can provide information on types of activities performed, duration and frequency. However, in older adults self-reporting evaluation of physical activity is subject to particular challenges, namely, due to changes in cognitive abilities and memory, which may lead to difficulties in understanding instructions on self-report measures and challenge their ability to recall their physical activity behaviours, especially over longer periods leading to less precise estimates [7]. Another aspect to consider is that in older age physical activity tends to be of lower intensity and highly variable, making the measurement of lighter activities essential in the elderly population, even though, many self report questionnaires are insufficient or inadequate to investigate these types of activities [8]. When framing these evaluations at an EU level they are also subject to social and cultural differences, as example, the very low educational levels and even high illiteracy of older adults in many EU regions, which creates a barrier to the use of self-completed questionnaires.

A review [15] showed 32 different self-report measures to assess physical activity in older adults, which could be divided into two broad groups of physical activity questionnaires (i.e., self- or interview administered questionnaires/surveys) or activity logs (i.e., records kept for a specified timeframe). The most commonly used self-report measures were the Physical Activity Scale for the Elderly (PASE), the Community Healthy Activities Model Program for Seniors Activities Questionnaire for Older Adults (CHAMPS) and activity diaries/logs (see table 1). These tools vary in their ability to quantify the frequency, intensity, type, and duration of various occupational, sports and leisure, transportation, and household activities over a variety of time frames.

### Objective measures of physical activity

As objective measures sensors are the most often used. The same review [15] showed six different types of direct measures for physical activity including accelerometers, pedometers, doubly labelled water, calorimetry, heart rate monitoring, and direct observation (see table 1). Objective measures are believed to offer more precise estimates of energy expenditure and remove many of the issues of recall and response bias. However, direct measures are more expensive, intrusive, time-consuming, and place a higher degree of burden on both the participant and the researcher than indirect measures [16, 18]. Also, individuals may alter their activity behaviour because they know it is being measured [19] Some measures (e.g., accelerometers, pedometers) provide very limited information about type of activity [20] and are not suitable for measuring certain types of PA (physical activity) (e.g., swimming, resistance exercise, upper body movements, cycling, complex movements; [16–20].

## WHAT TOOLS AND TECHNIQUES TO USE AND HOW TO MEASURE PHYSICAL CAPACITY ?

Physical capacity can be assessed using self-report measures or by objective measures, Table 2 provides an overview of the most commonly used measures to asses physical capacity. As with physical activity, the main advantages of subjective measures, are that they are cheap, easy-often self-administered, allow large sample size and have relatively low burden for the participants. The disadvantages are the limited scope, once they often only focus on only a subset of activities and when of self-administration the recall bias. Objective tests have the advantage of being independent of a subjective impression, most of them are very easy, quick to perform and require minimal requirement, although there are several different protocols for each objective dimension of physical capacity. An example of this is the chair stand test, one protocol is based on the number of times a subject sits and stands in 30 seconds while a another is based on the time a subject takes to sit and stand 5 times. Opting for a specific protocol should always be based on the studied elderly population, namely their predicted functional capacity, age, and co-mobility’s, once some of these protocols are validated for specific pathologies commonly present in older adults. The main disadvantage of these tests is that to perform them often trained professionals are needed and the outcomes dependent frequently on individuals motivation to perform maximally effort (6 minute walking test) or body characteristics (sit and reach test).

## IV HOW TO MAKE CHOICES ON WHAT TO USE FOR MEASURING PHYSICAL ACTIVITY OR PHYSICAL CAPACITY ?

**Figure 1:**
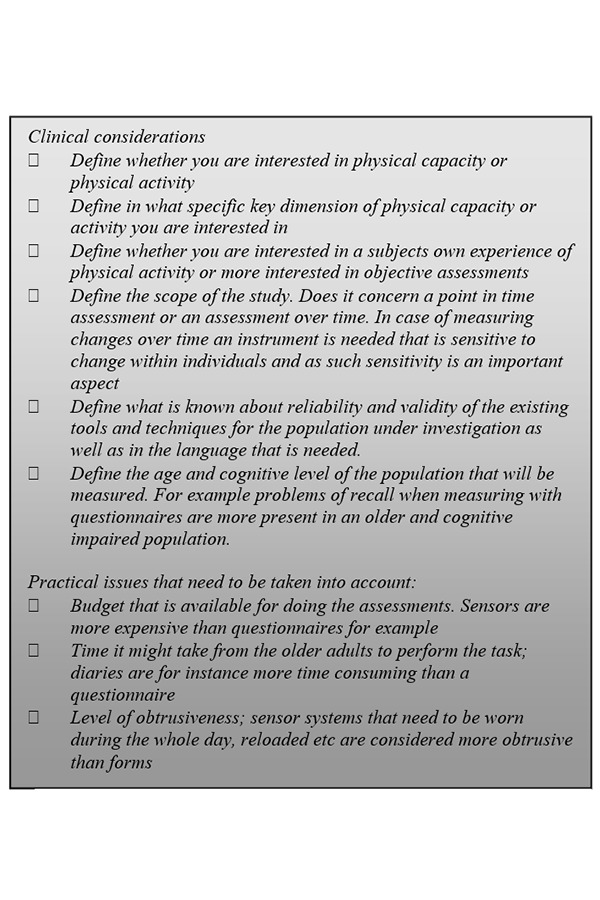
Checklist for deciding on concrete tools and techniques for assessing physical activity/physical capacity in older adults

From abovementioned overview it becomes clear that there are many tools and techniques to assess physical activity and physical capacity in older adults and there is no evidence to indicate which instruments are the more valid and reliable to use with older people [21] (as they measure different constructs). However, in order to support clinicians/researchers to come to a good choice, the checklist shown in figure 1 can be used as a starting point.

## V. EXAMPLES OF PHYSICAL ACTIVITY PROJECTS PERFORMED WITHIN THE UMBRELLA OF THE EIP-AHA A3 GROUP

In the following section an illustration of the state of play within the EIP-AHA A3 framework regarding physical activity in older adults is presented. For this two examples, a FP7 European project called PERSSILAA as well as an Italian local initiative called “Your Health into Movement”, are chosen. The PERSSILAA project was chosen as an example on how to select tools and techniques for assessing physical activity in older adults at a large scale across Europe. Also, with this example we aim to show the large variability in the scores on different physical capacity dimensions among older adults. Based on these aspects intervention strategies need to be adapted to the each individual, in order to understand this project “Your Health into Movement” has been selected.

### PERSSILAA

Bearing in mind the importance of preventing older adults from getting physically inactive and by this preventing them from becoming frail, the European project PERSSILAA (FP7-ICT-610359; www.PERSSILAA.eu) focused on the development of services for older adults to self-manage their physical activity behaviour. These services consisted of a self screening tool to be performed by each older adult every year and a self management training programme they can follow in case their physical capacity is declined. In order to be able to deploy this in a larger scale these services were offered to older adults through technology support. Screening was conducted with self screening subjective instruments validated questionnaires. Besides physical domains, frailty, cognition and nutrition were also measured, so the chosen instruments had to be short. For the physical domain the ten questions of the SF-36, focussing on the physical functioning, also known as the physical functioning scale (PFS) was selected [22]. Subjects were also asked to complete the Katz Index of Independence in Activities of Daily Living (KATZ-ADL)[23]. Based on the results of this screening older adult were classified as either physically fit or having some physical decline. Because the aim of PERSSILAA was to provide older adults with functional decline with a self management training program, all subjects that after screening were identified as having physical decline were invited to perform a second face-to-face screening during which four physical test were performed: the timed up and go [24], chair stand [25], chair sit and reach [26] and two-minute step [27]. Output of these tests; balance, strength, flexibility and endurance were used to select the right set of exercises for the self management training program, based on the OTAGO exercise program, which consisted of a 3 month home-based program that encompasses balance, strength and flexibility exercises, with increased difficulty along the program.

Since September 2014 this screening and training service has been implemented in the community of two regions, both reference sites of the EIP-AHA (region Campania, Italy and region Twente, the Netherlands) [28].

In Netherlands self screening was sent to 1234 older adults, a total of 643 (52%) completed and returned first screening, of these 68 did not give authorization for their data to be used for scientific publication purpose. Thus results are based on the outcomes of 575 subject with a mean age of 69,5 ± 5,3 years, 53,7% of which were females. Results are presented in Table 3.

Subject’s physical functioning level was determined according to PFS scale norm-scores, those with scores over 60 were considered at a normal physical functioning level and those below as having functional decline. Although average results of the Netherlands group indicates that their physically fit, a total of 32% scored below the norm, In contrast and according to KATS norm scores, (normal level of physical functioning is determined by scores ≤ 2score and physical decline scores over 2) only 4 older adults (2 of each gender) had a physical decline. In this Study the PFS showed to be more sensitive in this study that KATS to measure the level of physical functioning of older, based on which current PERSSILAA screenings rounds has left KATZ questionnaire once it did not show added value to PFS.

Based on the outcomes of the first screening phase 101 pre-frail older adults were invited for the second screening face to face. A total of 78 (29 male and 49 female) accepted to participate and performed the 4 physical tests previously described in the Netherlands.

Using the same protocol also Campania region, Italy included 88 (13 male and 75 female) older adults participated in the second screening.

Results of both countries show that:

- 80,5% (82,8% ♂ & 79,2% ♀) of the Dutch and 83,3% (72,7% ♂ & 85,5% ♀) of the Italian sample, scored below norm in the timed up and go test showing a decrease in mobility and balance.
- 58,9% (70,4% ♂ & 52,2% ♀) of the Dutch and 32% (36,4% ♂ & 31% ♀) of the Italian sample scored below the norm in the sit and stand test showing a decrease in strength.
- 35,6% (30,8% ♂ & 38,3% ♀) of the Dutch and 79% (27,3% ♂ & 89,1% ♂) of Italians, scored below the norm in the sit and reach test manifesting a decreased flexibility.
- 15,7% (20% ♂ & 13,3% ♀) of the Dutch and 57,6% (45,5% ♂ & 60% ♀) of the Italians scored below the norm in the two-minute step test showing a decreased in endurance.

In both countries older adults who reported a physical decline in screening showed to have 1^st^ and 2^nd^ in order mobility and balance problems, which is followed by a decrease in strength, flexibility and endurance in the Netherlands whereas in Italy the order was flexibility, endurance and strength. After the second screening the Netherlands sample was invited to participate in a cohort multiple Randomized Controlled Trail (cmRCT) to evaluate the ICT supported OTAGO program. Results of the first RCT with 15 pre-frail older adults in the intervention group showed positive results in terms of acceptance, adherence to the program as well as on quality of life and health status. The results of this evaluation are currently being published.

### Your Health into Movement

“*Your Health into Movement*” is a local project implemented by the Department of Neurological, Biomedical and Movement Sciences of the University of Verona, in cooperation with Verona Municipality. The project aims to put into effect the concrete link among ageing – physical activity – healthy ageing, through the implementation of a model of applied research (position stand ACSM, 2011[11]) consisting of a multidisciplinary staff integrating community services that offers individualized physical activity programs and training and educational scope for the group of people over 55, with and without chronic diseases (hypertension, heart disease, COPD, metabolic diseases, post stroke, Parkinson’s disease). The project involves every year 400 people over 55, coming spontaneously or sent by medical doctor of the Hospitals of Verona, in case of specific diseases.

The program consists of a first interview for screening during which staff collects all personal information and medical documents, and a first evaluation with various objective test to assess physical capacity (6 min walking test, adapted Step Test, Sit & Reach, Back Scratch, 6 RM Leg Press, 6 RM Biceps Curl, 1 min Sit Up) and anthropometric measures (weight, height, BMI, waist and hip ratio (WHR)). In addition, the physical activity readiness questionnaire (PAR-Q) is completed. This is a self-screening tool, useful to determine the safety or possible risk of exercising for an individual based upon their answers to specific health history questions.

With the results of these tests a detailed insight is gained in the physical capacity of each individual in terms of strength, endurance and mobility, this detailed information is used to refer each individual older adult to the most tailored program. Table 4 explains the different activity programs that include cardiorespiratory exercise, resistance exercise, flexibility exercise and neuromotor exercise, organised in 3 days/week of training, from September to July. At the end of the 11-month-program each subject is assessed again using the same test as before and each subject receives an individual evaluation report comparing the tests performed at the beginning and at the end of the program. The aim of this reports is to inform people on performance, on the capacities trend, and increase one’s awareness on personal health status after training. From September 2014 to July 2015, 400 old people were involved in specific training programs whereof the 20% was in a good health status and 80% with chronic diseases. General attendance over this period was about 70% of the prescribed sessions, even if a part of participants were very often in a non stable health condition.

#### Discussion

Due to the fact that physical activity level is related to and therefore very important for healthy aging, physical activity is getting more and more attention as a way of improving aging, however physical activity in elderly is very diverse and scattered. The aim of this paper was to give clinicians, researchers, policy makers and other interested stakeholders some insight in how physical activity can be defined, parameterized and measured in older adults and also give some insights on how to deal with physical activity promotion among elderly citizens at a European level.

From this paper three important aspects should be highlighted:

1] When talking about physical activity two different aspects are often unfairly mixed up namely physical activity and physical capacity. These are two different aspects that complement each other but are both important to get insight in someone’s physical functioning status.
  Quite recently a third term aspect appeared to be important in this respect “sedentary behaviour” [29,30]. Sedentary behaviour refers to any waking activity characterized by an energy expenditure ≤ 1.5 metabolic equivalents and a sitting or reclining posture [31]. In general any time a person is sitting or lying down, they are engaging in sedentary behaviour. Common sedentary behaviours include TV viewing, video game playing, computer use (collective termed “screen time”), driving automobiles, and reading. Sedentary behaviour is called the new smoking as too much time spent sitting is an independent risk factor for obesity and metabolic health problems *separate and distinct from getting too little exercise*. As this insight is relatively new, none of the norms presented in these papers has explicit recommendations for the time someone is allowed to spend sedentary maximally. This should be added in our opinion.
2] Both physical activity and physical capacity can be expressed in different dimensions like time, frequency, type of activity with the consequence that there are many instruments and protocols available, all of which have advantages and disadvantages. There is no clear evidence to indicate which instruments are the more valid and reliable to use with older people as they measure different constructs which makes it impossible to come with concrete recommendations on what to use. However we were able to define a set of 9 criteria that can support people to choose an appropriate instrument in their every day practice.
3] When putting into practice it appears, shown by the PERSSILAA case, that older adults score differently across the different physical functioning dimensions, that there are differences between individual older adults and probably also differences between groups of older adults from different countries. It appears that of a community dwelling population over the age of 65 around 32% experience limitation in physical functioning and in most of these cases (around 80%) there is at least a decline in mobility and balance. Giving this individual difference, strategies should also be adapted to the individual abilities. The “Your Health into Movement” case is one of the striking examples that indicates how exercise programs can be developed taking into account the individual variability.

Finally, looking a bit in how in future technologies will further emerge and may be more and more adopted by older adults. It is believed that technology will greatly enhance the field of physical activity promotion both in terms of assessment, as well as, for offering personalized intervention strategies [32,33]. Consider for example the possibility to unobtrusively monitor physical activity through environmental sensors, sensors integrated in mobile devices and/or on body sensors which provides detailed insight in individual profiles but also in small changes in some ones activity pattern (probably without being noticed by the individual themselves). Having access to this information will give researches the possibility to deliver individual interventions/coaching strategies to each subject or to whoever supports them in keeping physically active. Technology, can also be used to deliver complete exercise programs into home environments (home-based programs), and can be used to help monitor progression and further tailored when needed.

## Figures and Tables

**Table 1:** Overview of the most commonly used instruments to measure physical activity

**Type of method**	**Instrument**	**Key dimensions**	**Disadvantages**	**Advantages**
**Subjective**	**PASE**	Occupational, household and leisure items	Difficult to score; Takes some time to fill in; problem with recall (1 week)	Inexpensive, allowing large sample size, low participant burden, easy-administered
	**CHAMPS**	Assesses weekly frequency and duration of various physical activities typically undertaken by older adults.	Problems of recall of activity (4 weeks); takes some time to fill in	Inexpensive, allowing large sample size, low participant burden, easy-administered
	**Activity diaries/logs**		Problems with recall of activity, especially in the older adult Potential content validity problems associated with misinterpretation of physical activity in different populations/countries	Inexpensive, allowing large sample size, low participant burden, easy-administered
**Objective**	**Activity monitors**	Movement counts	Some might be obtrusive; some are expensive, inaccurate assessment of a large range activities (eg upper body movement, cycling, water-based activities)	Objective measure of bodily movement; useful in field settings, non-invasive, allows for extended period of recording (weeks, months); potential to promote behaviour change
	**Pedometers**	Step counts	Are specifically designed to assess walking only; loss of accuracy when jogging or running	Inexpensive, non-invasive, easy to administer, potential to promote behaviour change
	**Doubly labelled water and indirect calorimetry**	Energy expenditure	Expensive; invasive	Very precise
	**Heart rate monitoring**	Beats per minute	Obtrusive, expensive when used for large numbers of participants	Valid in field settings, easy and quick data collection
	**Direct observation**	Activity rating	Time consuming; observer presence might alter normal physical activity	Provides excellent quantitative and qualitative information

**Table 2 :** **Overview of the most commonly used instruments to measure physical capacity**

**Type of method**	**Instrument**	**Key dimensions**
**Subjective**	**KATZ index**	Assesses basic activities of daily living: bathing eating dressing continence transfers to toilets locomotion
	**(modified) Barthel index**	Feeding urinary and faecal continence personal toilet dressing toilet use transferring walking outdoors climbing stairs bathing
	**Health assessment questionnaire**	Dressing rising eating walking hygiene reach grip activities
	**Physical functioning scale SF36**	Vigorous activities, moderate activities, lifting or carrying groceries, climbing several flights of stairs, bending, kneeling or stooping, walking more than one mile, walking several blocks
**Objective**	**Short Physical performance Battery (SPPB)**	The SPPB captures is a combination of tests testing lower extremity Test cover the domains: - strength (sit to stance test) - walking velocity (4 meter walking test) - balance (tendom test)
	**Sit and stand**	Lower limb strenght
	**Walking tests**	Walking velocity
	**Timed up and go**	Mobility Static and dynamic balance
	**Sit & Reach**	Flexibility
	**6 minute walking**	Walking velocity, predicter of cardiovascular Endurance
	**Adapted Step**	Cardiovascular fitness
	**6RM**	Predictor for maximum
	**Biceps Curl**	stenght of the elbow flexors
	**Back Scratch**	General shoulder range of motion
	**6 RM leg Press**	Lower limb strenght
	**Hand Grip**	Hand flexors strengh

**Table 3:** Overview of the physical outcome of the first screening PERSSILAA service module region Twente, the Netherlands (mean and standard deviation (SD)).

	Total group (n=575)	Male (n=268)	Female (n=307)
Physical functioning scale (PFS SF-36	71,6 ± 28,6	77,2 ± 27,7	66,7 ± 28,4
KATZ-ADL	0,3 ± 0,7	0,2 ± 0,7	0,3 ± 0,0

**Table 4:.** **Summary of Physical Activity Interventions for older adults in the project “Your Health into Movement”**

**Physical Activity**	**Frequency (F)**	**Intensity (I)**	**Duration (D)**	**Adaptation for chronic disease**	**Examples**
Endurance exercise	3 d/wk	60–75% HRR	20–30 min (in bouts of at least 10 min each)	People with beta-blockers, Intensity is measured by the Rating of Perceived Exertion or Borg scale (scale 6–20). I: 12–14 RPE Hypertension: I: 40–60% HRR, D: 40 min Diabetes: I: 50–70% HRR, D: 40 min Stroke: I: 50–80% HRR, D: 20-20-40 min Osteoarthritis: I: 40–60% HRR	Walking, jogging and cycling
Resistance exercise	3 d/wk	60–70% 1RM	4–5 exercises involving the major muscle groups, trained 3 sets of 8–12 repetitions each	Osteoarthritis: isometric exercises are included	Traditional isotonic exercise (Leg press, Chest Press, Lat Machine, …)
Flexibility exercise	3 d/wk	Moderate (5–6) intensity on a scale of 0 to 10	10 exercise involving the major muscle groups, 30 s each		Static stretch and exercise performed in full R.O.M.
Balance exercise	3 d/wk	(N.A.)	Al least 30 s for 6 different positions		balance board exercises

## References

[1] The Ageing Report 2015. European Economy.

[2] Chodzko-Zajko W, Schwingel A, Hee Park C (2009). Successful Aging: The Role of Physical Activity. American Journal of Lifestyle Medicine.

[3] Tribess S, Júnior J, Oliveira R (2012). Physical activity as a predictor of absence of frailty in the elderly. Elsevier Editora Ltda.

[4] Howe Tracey E (2007). “Exercise for improving balance in older people.”. The Cochrane Library.

[5] Liu Chiungju, Latham Nancy K (2009). “Progressive resistance strength training for improving physical function in older adults.”. The Cochrane Library.

[6] Sun Fei, Norman Ian J, While Alison E (2013). “Physical activity in older people: a systematic review.”. BMC public health.

[7] DiPietro L (2001). “Physical activity in aging: changes in patterns and their relationship to health and function.,”. J. Gerontol. A. Biol. Sci. Med. Sci.

[8] Forsén Lisa (2010). “Self-administered physical activity questionnaires for the elderly.”. Sports Medicine.

[9] Caspersen CJ, Powell KF, Christenson GM (1985). Physical activity, exercise and physical fitness: definitions and distinctions for health-related research. Public Health Rep.

[10] Garber CE, Blissmer B, Deschenes MR, American College of Sports Medicine Position Stand (2011). Quantity and quality of exercise for developing and maintaining cardiorespiratory, musculoskeletal, and neuromotor fitness in apparently healthy adults: Guidance for prescribing exercise. Medicine & Science in Sports & Exercise.

[11] (2010). Global Recommendations on Physical Activity for Health. http://www.ncbi.nlm.nih.gov/books/NBK305057/.

[12] Kemper H, Ooijendijk W, Stiggelbout M (2000). Consensus over de Nederlandse Norm voor Gezond Bewegen. [Consensus on the Dutch Norm for Healthy Physical Activity.]. Tijdschr Soc Gezondheids.

[13] Physical Activity Guidelines Advisory Committee (2008). Physical Activity Guidelines Advisory Committee Report.

[14] S. Department of Health and Human Services, Physical Activity Guidelines Advisory Committee (2008). Physical Activity Guidelines Advisory Committee Report 2008.

[15] Kowalski K, Rhodes R, Naylor P-J, Tuokko H, MacDonald S (2012). “Direct and indirect measurement of physical activity in older adults: a systematic review of the literature.,”. Int. J. Behav. Nutr. Phys. Act.

[16] Adamo KB, Prince SA, Tricoo AC, Connor-Gorber S, Tremblay M (2009). A comparison of indirect versus direct measures for assessing physical activity in the pediatric population: A systematic review. Int J Pediatr Obes.

[17] Dale D, Welk GJ, Matthews CE, Welk GJ Physical activity assessments for health-related research.

[18] Prince SA, Adamo KB, Hamel ME, Hardt J, Gorber SC, Tremblay M (2008). A comparison of direct versus self-report measures for assessing physical activity in adults: a systematic review. Int J Behav Nutr Phys Act.

[19] ilcox S, Ainsworth BE, Shumaker SA, Ockene JK, Riekert KA (2009). Chapter 17. The measurement of physical activity. The Handbook of Health Behavior Change.

[20] Murphy SL (2009). Review of physical activity measurement using accelerometers in older adults: considerations for research design and conduct. Prev Med.

[21] Garatachea N, Torres Luque G, Gonzalez Gallego J (2010). “Physical activity and energy expenditure measurements using accelerometers in older adults.”. Nutr Hosp.

[22] Ware JE AA, Snow KK, Kosinski M, Gandek B (1989). SF-36 Health Survey: Manual and Interpretation Guide.

[23] Katz S, Ford AB, Moskowitz RW, Jackson BA, Jaffe MW (1963). Studies of Illness in the Aged. The Index of Adl: A Standardized Measure of Biological and Psychosocial Function. JAMA.

[24] Morris S, Morris ME, Iansek R (2001). Reliability of measurements obtained with the Timed “Up & Go” test in people with Parkinson disease. Phys Ther.

[25] Jones CJ, Rikli RE, Beam WC (1999). A 30-s chair-stand test as a measure of lower body strength in community-residing older adults. Res Q Exerc Sport.

[26] Różańska-Kirschke AK, P, Wilk M, Dylewicz P (2006). The Fullerton Fitness Test as an index of fitness in the elderly. Medical Rehabilitation.

[27] Rikli RE, Jones CJ (1999). Functional fitness normative scores for community-residing older adults, ages 60–94. Journal of Aging and Physical Activity.

[28] van Velsen L A Community-Based, Technology-Supported Health Service for Detecting and Preventing Frailty among Older Adults: A Participatory Design Development Process. Journal of Aging Research.

[29] Maher JP, Conroy DE (2015). Health Psychol.

[30] Song J, Lindquist LA, Chang RW, Semanik PA, Ehrlich-Jones LS, Lee J, Sohn MW, Dunlop DD (2015). Sedentary Behavior as a Risk Factor for Physical Frailty Independent of Moderate Activity: Results From the Osteoarthritis Initiative. Am J Public Health.

[31] Mansoubi M, Pearson N, Clemes SA, Biddle SJ, Bodicoat DH, Tolfrey K, Edwardson CL, Yates T (2015). Energy expenditure during common sitting and standing tasks: examining the 1.5 MET definition of sedentary behaviour. BMC Public Health.

[32] Silveira P1, van de Langenberg R, van Het Reve E, Daniel F, Casati F, de Bruin ED (2013). Tablet-based strength-balance training to motivate and improve adherence to exercise in independently living older people: a phase II preclinical exploratory trial [1]. J Med Internet Res.

[33] Delbaere K, Valenzuela T, Woodbury A, Davies T1, Yeong J, Steffens D, Miles L, Pickett L, Zijlstra GA, Clemson L, Close JC, Howard K, Lord SR (2015). Evaluating the effectiveness of a home-based exercise programme delivered through a tablet computer for preventing falls in older community-dwelling people over 2 years: study protocol for the Standing Tall randomised controlled trial.. BMJ Open.

